# Can Monoclonal Antibodies against CGRP Offer New Treatment Options for Type 2 Diabetes?

**DOI:** 10.33696/diabetes.1.028

**Published:** 2020

**Authors:** Celine E Riera

**Affiliations:** 1 Center for Neural Science and Medicine, Department of Biomedical Sciences, Board of Governors Regenerative Medicine Institute, Department of Neurology, Cedars-Sinai Medical Center, 127 South San Vicente Boulevard, Los Angeles CA 90048, USA; 2 David Geffen School of Medicine, University of California, Los Angeles, USA

The neuropeptide Calcitonin Gene-Related Peptide (CGRP) is a 37-amino acid peptide, with a wide-range of biological activities including vasodilation [1], neurogenic inflammation [1], immune function [2] and hypertension [3]. In addition to these various roles, it has also been heavily implicated in metabolic disease, with roles in feeding, energy dissipation processes and pancreatic β-cell insulin secretion. One of the most striking effects of delivering CGRP either by intraperitoneal or intracranial routes is an acute reduction of food intake and energy expenditure [4–7]. This important function has been linked to activation of brain parabrachial neurons which contain CGRP and acutely suppress feeding to cause starvation [8]. Remarkably, CGRP is present in both central and peripheral nervous systems, where it is likely to have different biological activities. Krahn et al. noted that intracranial CGRP delivery was more potent at inhibiting feeding compared to intraperitoneal route [5]. Moreover, whole-body deletion of mouse CGRPα increased food intake, but also led to a surprising resistance to weight gain on diet-induced obesity [9], suggesting that complementary effects on energy expenditure were being recruited to dissipate the additional calories ingested. These data highlight the need to scrutinize central and peripheral specificity of the CGRP peptide in energy balance.

Evidence that CGRPα may have dedicated function in the pancreas comes from a wide number of early studies demonstrating a potent role in antagonizing insulin release from pancreatic β-cells [10–16]. In the pig, intrapancreatic infusion of picomolar concentrations of CGRP was sufficient to reduce glucose-stimulated insulin secretion (GSIS) by 45% and increased glucagon secretion by 7-fold [10]. Similar inhibition of GSIS was observed in intravenous delivery of CGRP in the rat [15]. Correlative evidence demonstrates that loss of sensory fibers containing CGRP are associated with increased insulin secretion and improved glucose tolerance [12,16,17]. Similarly, inactivation of the sensory receptor TRPV1, which functions upstream of CGRP in nociceptive sensory neurons, is associated with increased insulin secretion [18,19] and resistance to diet-induced obesity [20].

Recently, monoclonal antibodies have been developed against CGRP to treat migraines and effectively reduce the frequency of attacks in chronic and episodic migraine patients [21]. This peptide is abundant in the trigeminal ganglion and due to its important role as a vasodilator, its function in migraine is likely mediated by promoting neurogenic inflammation of meningeal vessels which drives peripheral and central pain sensitization [22]. Several antibodies have reached approval by the FDA and include eptinezumab (ALD403), erenumab (AMG 334), galcanezumab (LY2951742), and fremanezumab (TEV-48125) [23]. Due to their large size, these antibodies do not cross the blood brain barrier [21], and have long half-lives. Monoclonal CGRP antibodies (mAbs) provide a potential strategy to tease apart the peripheral versus central role of CGRP in glycemic control and energy metabolism.

Earlier findings highlighting the ability of CGRP to reduce insulin secretion are further supported by data from the recent report of Halloran et al. demonstrating that monoclonal therapy against CGRP specifically improved glucose tolerance and insulin resistance in monogenic models of diabetes in mice [24]. Mice homozygous for the Lepr^db^ mutation (db/db) lack the leptin receptor and display hyperphagia, obesity and type 2 diabetes. In the C57BL/6J background, these mice manifest transient hyperglycemia, pancreatic β cell hypertrophy and hyperinsulinemia [25]. Mechanistically, Halloran et al. found that GSIS was increased with CGRP mAb antibody treatment in the db/db animals (C57BL/6J strain). Importantly, mAb treatment did not modulate food intake, consistent with lack of penetrance of this antibody in the brain. The treatment also ameliorated glucose tolerance in ob/ob mice (Lepr^ob^ mutation), which lack leptin and develop hyperglycemia and β cell hypertrophy. In accordance with a β cell-dependent inhibition of insulin release by the CGRP peptide, monoclonal CGRP therapy failed to restore normoglycemia in streptozotocyn-mediated loss of pancreatic islets. Direct evidence of CGRP ability to block GSIS was provided in functional assays to measure GSIS in primary mouse islets, where recombinant CGRP peptide blocked GSIS compared to vehicle treatment.

A key observation in the study by Halloran et al. was the inability of the CGRP mAb to ameliorate glucose tolerance and insulin sensitivity in mice fed a high fat diet and manifesting diet-induced obesity. This drastic difference in response, compared with the monogenic models of diabetes, may arise from divergent pancreatic islet plasticity between Lepr^db^ and wild-type BL6 mice. β-cell hypertrophy is highly dependent on the mouse strain and intervention performed. It is well established that weight gain due to high fat diet feeding leads to increase insulin secretion combined with fast expansion of β-cell mass to ramp-up insulin production [26,27]. β-cell proliferation is the primary method by which β-cell mass increases during diet-induced obesity, stimulated by increased blood insulin and glucose concentrations. Since the CGRP mAb failed to ameliorate glucose tolerance in high fat fed mice, possible rapid β-cell expansion may have blunted the effects of the antibody. In contrast, the diabetic db/db strain in BL6 background, which responded well to mAb treatment is well characterized to develop transient hyperglycemia and slower β-cell hypertrophy [25]. Interestingly, in the BKS background, the antibody improved Lepr^db^ glycemia but had minor effects on body weight (unpublished observations). The Lepr^db^ mutation leads to different forms of diabetes depending on the animal’s genetic background and is characterized by severe depletion of the pancreatic β-cells in the BKS mice and a more severe diabetic outcome, whereas compensatory hyperplasia is seen in BL6 mice together with increased survival [28]. The ability of this treatment to improve glycemia in these two strains suggests that reduction of secreted CGRP levels is beneficial to diabetic mice presenting moderate changes in islet mass during early stages of the disease. CGRP’s ability to block β-cell GSIS may be a significant contributor to diabetes onset in these monogenic models. Together with circulating CGRP levels, transcript levels of calcitonin receptor like receptor (*Calcrl*) and receptor activity-modifying protein 1 (*Ramp1*), which encode for the CGRP receptor, are enhanced in isolated Lepr^db^ islets. The presence of a maladaptive CGRP-dependent signaling in response to weight gain, aging or other conditions associated with impaired insulin secretion from β-cells could promote hyperglycemia [3,29–31]. Mechanistically, it remains unknown whether CGRP’s action on β-cells is the sole contributor to hyperglycemia, or whether other biological functions of this peptide such as vessel dilation may also participate in disease establishment.

Additionally, the peripheral mechanism of action of CGRP mAb to reduce glucose levels and weight gain may be due to combined modulation of GSIS as well as loss of adiposity in the mice. High fat feeding-dependent weight gain and increased adiposity was significantly reduced upon CGRP mAb treatment. Halloran et al. provide further evidence that CGRP modulates adipose tissue energy utilization in accordance with a previous study demonstrating a causal link between CGRP and lipolysis in soleus muscle [32]. Gene expression of fatty-acid oxidation related transcripts, as well as ex-vivo lipolysis was improved in diabetic mice treated with CGRP mAb. Additionally, CGRP application on primary adipocytes reduced palmitate-dependent β-oxidation. Therefore, the improved energy expenditure phenotype manifested by mice receiving this mAb is likely to be derived from improved adipose lipolysis and energy utilization. These phenotypes are reminiscent of the leaner profile of CGRP knockout mice on DIO [9].

Can monoclonal antibodies against CGRP be considered in the treatment of type 2 diabetes? This prospect needs to be considered in the context of the pharmacokinetics of CGRP mAbs and their potential side effects on liver and cardiovascular physiology. Blocking CGRP in migraine patients is well tolerated, but the abundant presence of CGRP receptors in the vasculature may pose a risk for subjects presenting comorbidities, such as type 2 diabetes and cardiovascular diseases. Initial studies on inhibiting the CGRP pathway with small molecules induced liver toxicity, but mAbs do not produce toxic metabolites [21,33]. The potential long-term effects of blocking CGRP are not known, but a major advantage of these antibodies stems from their long half-life allowing monthly or less frequent injections, which can remarkably improve adherence to the treatment and its following effectiveness [21,33]. Thanks to their long half-life, current mAbs are given through monthly subcutaneous administration (Fremanezumab, Galcanezumab, Erenumab) or intravenous delivery every 3 months (eptinezymab). Yet, pharmacokinetic properties of these mAbs remains poorly understood, with estimation that a single injection may neutralize circulating CGRP for 6–7 weeks [34].

Despite the theoretical harmful inhibition of vasodilation due to CGRP inhibition, no cardiovascular concerns have yet been disclosed with any of these mAbs. Remarkably, CGRP-positive perivascular nerves innervate mostly small blood vessels, and are weakly present around epicardial coronary veins of the heart [35,36]. CGRP has differential effects on coronary arteries: small relaxation of the proximal portions and large vasodilation of the distal portions, which also are more densely innervated with CGRP-containing fibers [34]. CGRP has been shown to exhibit protective action against cardiovascular insults, including ischemic events and tissue remodeling in pulmonary hypertension [34]. This protective action is concerning since diabetic patients present an increased cardiovascular risk [37]. Many vascular disorders are strongly associated with diabetes, including retinopathy and nephropathy, peripheral vascular disease, stroke, and coronary heart disease [37]. On the other hand, plausible beneficial outcomes are likely to arise by delivering mAb treatment to diabetic patients and lead to reduced progression of cardiovascular morbidity by lowering hyperglycemia, which contributes to diabetic microvascular disease. Small vessel damage, including retinopathy, nephropathy and neuropathy are associated with high cardiovascular mortality. However, neuropathy is correlated with reduced bioavailability of nitric oxide (NO), a potent vasodilator and a state of vasoconstriction which favors hypertension [38,39]. Therefore, blocking CGRP, another strong vasodilator, may further aggravate the pre-existing hyper-constricted state of the vasculature in diabetes caused by reduced NO levels. It is unknown whether blocking CGRP could potentially increase cardiovascular complications and whether these risks are higher in certain patients [34]. Women, who present higher levels of plasma CGRP, could be at increased risk but studies targeting mAb safety in women cardiovascular health are still missing [34].

An additional drawback of antibody therapies is the risk of developing antidrug antibodies, which impairs efficacy. Indeed, antidrug antibodies immunogenicity was detected with all four antibodies but these did not seem to affect efficacy [33,40,41]. Nevertheless, long-term studies are needed to investigate whether neutralizing antidrug antibodies will pose a problem for effectiveness and safety of blocking CGRP with monoclonal antibodies.

Finally, when contemplating a new medication to a disease with already many therapeutic options, one must evaluate if the CGRP mAb could provide any benefit to already existing drugs. Many pharmaceutical options are available to lower blood glucose and achieve their effects through successful modulation of one or several biological pathways leading to hyperglycemia. Recognized molecular targets for type 2 diabetes treatment include elevating insulin secretion from pancreatic β-cells; increasing glucagon secretion from pancreatic α cells; boosting liver glucose production; management of brain insulin resistance; increasing lipolysis; enhancing renal glucose reabsorption; lowering incretin effect in the small intestine; and mitigating the impaired or diminished glucose uptake in peripheral tissues such as skeletal muscle, adipose tissue and liver [42]. According to currently available rodent research, the CGRP mAb could act on two of the pathways described above and benefit diabetic patients to improve insulin secretion and lipolysis.

Out of the major classes of oral antidiabetic medication, drugs capable of overlapping action are incretin mimetics, such as glucagon-like peptide (GLP-1) and its analogs [43]. The GLP-1 and glucose-dependent insulinotropic polypeptide (GIP, or incretin) hormones work as insulin secretagogues to reduce blood glucose levels. Because these peptides have a short half-life and are degraded by the dipeptidyl peptidase-4 (DPP-4) enzyme, molecule analogs and DPP-4 inhibitors have both been developed. The American Diabetes Association recommends the usage of GLP1 analogs or DPP-4 inhibitors as alternatives or in combination with metformin when glycemic levels remain higher than normal [44]. Metformin is the most widely used antihyperglycemic and the preferred pharmacological agent for type 2 diabetes treatment. Metformin’s primary mechanism of action lies in its ability to reduce hepatic glucose production, and lower glucose through mild enhancement of insulin-stimulated glucose uptake [42]. CGRP mAb may present some benefits as alternate therapy to incretin-based drugs, which must be administered after every meal or through daily injections to remain effective. The side-effects of incretin-based treatments are relatively mild but nonetheless include upper respiratory tract infections, nausea, headache and hypoglycemia in some cases. However, incretins may offer protective actions on cardiac function in patients with ischemic heart disease [43,45], a condition that might be worsened by the CGRP mAb [34]. In comparison with incretin drugs, the CGRP mAb therapy could be more costly but could require fewer injections to achieve its effects. Further studies investigating this antibody in human diabetes are necessary to evaluate its blood glucose lowering potency in comparison with incretins. It will be important to determine whether it can function as a co-treatment with metformin and assess its ability to limit diabetic complications.

## Conclusions

Taken together, the current evidence supports the use of CGRP mAb in pre-clinical model of type 2 diabetes, with successful management of blood glucose in animal models of monogenic diabetes. Further studies are necessary to evaluate the ability of this treatment to lower blood glucose in human type 2 diabetes. In particular, it will be fundamental to clarify the significance of this treatment in cardiovascular complications and potential side-effects that may arise in diabetic subjects. Type 2 diabetes mellitus is a chronic condition with multiple treatment options, combined with lifestyle interventions. Improving treatment efficiency, cost and frequency of administration is a priority with the number of patients steadily increasing world-wide.
